# The National Insurance Bill

**Published:** 1911-05-20

**Authors:** 


### THE NATIONAL INSURANCE BILL.

The National Insurance Bill contains eighty-seven clauses and nine schedules, but it is not necessary to add much to the general explanatory notes of the scheme which appeared in The Hospital last week. The position of medical practitioners and hospitals in relation to the scheme, however, requires further consideration. In elaborating the proposals the Chancellor gave the impression that a fixed fee had been arranged for medical practitioners, but this impression was erroneous, for Clause 14, which deals with the administration of medical benefit, makes no such provision. The first two paragraphs of this clause are as follows:—

1. Every approved society and local health committee shall, for the purpose of administering medical benefit, make arrangements with duly qualified medical practitioners for insured persons to receive attendance and treatment to the satisfaction of the Insurance Commissioners from such practitioners.

2. Every such society or committee shall also make provision for the supply of proper and sufficient drugs and medicines to insured persons, and no arrangement shall be made with a medical practitioner under which he is bound or agrees to provide drugs or medicine for any insured person at an inclusive fee, without the consent of the Insurance Commissioners, which consent they shall not give unless the circumstances of any locality situate in a rural district are such as to make it expedient to do so.

It will be observed that it is left to the Friendly Societies to make terms with medical practitioners, and already the officials of the societies are speaking of a fee of 4s. per member per annum as generous terms. Experience has shown that Friendly Societies are not always amenable to reason when making arrangement for medical attendance on their members, and an attempt should, and no doubt will, be made to amend the first paragraph of Clause 14 in such a way as to prevent these societies from distributing charity at the expense of the medical profession. It was pointed out last week that it was not clear that the maternity grant of 30s. was going to the practitioner and the nurse. The text of the Bill does not clear up this point altogether, but the second paragraph of Clause 16 provides that

"The money payable by way of maternity benefit shall be expended in such a manner as may be prescribed, and no payment shall be made by way of maternity benefit if the mother of the child has failed to comply with such conditions as may be prescribed."

If the mother of the child is herself an insured person maternity benefit is to be treated as a benefit for her, but if she is not a member of any society, by the local health committee; if she is not herself an insured person the benefit is to be treated as a benefit for her husband and is to be payable in respect of a posthumous child. Provision is made in Clause 12 in the case of contributors who are inmates of hospitals. This clause provides that no payment shall be made in respect of sickness, disablement, or maternity benefit to any insured person during any period when he is an inmate of any workhouse, hospital, asylum, or infirmary supported by any public authority, or out of any public funds, or by a charity, or of a sanatorium or similar institution established under the Bill. During such period any benefit which would otherwise have been payable to such person is to be applied for the relief of his dependants, but if he is an inmate of a sanatorium and has no dependants the benefit is to be paid to the local health committee. Apparently no provision is made for the payment of the benefit to hospital authorities, but Clause 17 gives Friendly Societies power to subscribe to hospitals. The clause is as follows :—

"It shall be lawful for any approved society to grant such subscriptions or donations as it may think fit to hospitals and other charitable institutions, or for the support of district nurses, and to appoint nurses for the purpose of visiting insured persons who are members of the society, and any sums so expended shall be treated as expenditure on such benefits under this part of the Act as may be prescribed."

The Insurance Commissioners, with a central office in London, are to be appointed by the Treasury; the qualification of Commissioners is not stated in the Bill, but it is understood that medical men are to be represented. There is also to be an Advisory Committee for the purpose of giving the Commissioners advice and assistance in connection with the making and altering of regulations; this committee is to be appointed by the Commissioners. On local health committees there are to be at least two duly qualified medical practitioners. One of the duties of local health committees will be to make provision for the giving of lectures and the publication of information on questions relating to health, and in carrying out their duties the committees are to have the assistance of county and borough medical officers of health.

